# One-Minute Silent Video Clips: A Database of Valence and Arousal

**DOI:** 10.5964/ejop.14685

**Published:** 2025-08-29

**Authors:** Vladimir Kosonogov, Kirill Efimov, Olga Kuskova, Isak B. Blank

**Affiliations:** 1 Affective Psychophysiology Laboratory, Institute of Health Psychology, HSE University, Saint Petersburg, Russian Federation; 2 Institute for Cognitive Neuroscience, HSE University, Moscow, Russian Federation; Università Cattolica del Sacro Cuore, Milan, Italy

**Keywords:** emotion, valence, arousal, video, database

## Abstract

Researchers in the behavioral and social sciences use linear discriminant analysis (LDA) for predictions of group membership (classification) and for identifying the variables most relevant to group separation among a set of continuous correlated variables (description). In these and other disciplines, longitudinal data are often collected which provide additional temporal information. Linear classification methods for repeated measures data are more sensitive to actual group differences by taking the complex correlations between time points and variables into account, but are rarely discussed in the literature. Moreover, psychometric data rarely fulfill the multivariate normality assumption.

The article introduces a dataset consisting of 160 one-minute affective video clips with normative values of valence and arousal. Each video was evaluated by 30 subjects, while each subject evaluated at least 20 videos. Compared to previous attempts to collect affective videos, the dataset has several advantages. Firstly, the high number of videos in different valence categories allows researchers to compile appropriate subsets for their studies. Secondly, the approximately equal and conventional duration of videos makes it possible to use them in psychophysiological studies applying EEG, fMRI, peripheral polygraphy, posturography, TMS, etc. Thirdly, the exclusion of sound or speech that might provoke culture-dependent interpretation makes the dataset useful in different cultures. The relationship between valence and arousal showed a typical quadratic pattern, with very negative and very positive videos receiving higher levels of arousal. Several negative videos received greater arousal scores than the most positive ones, reflecting negativity bias. The dataset encompasses more than 50 videos of different valence (negative, neutral, and positive ones). We believe that it will permit researchers to select corresponding subsamples of videos from different categories for their studies.

In the field of emotion research, affective stimuli in the form of images, words, and video clips are frequently employed. The availability of these stimuli sets is constantly increasing, leading to a vast number of options for researchers to choose from. This can pose a challenge for those seeking the most suitable stimuli for their studies. However, ecologically valid media-based stimuli, like video clips, can offer meaningful insights into the natural progression of mental processes, including emotion regulation and speech perception (Jääskeläinen & Kosonogov, 2023). Additionally, research indicates that naturalistic paradigms, like presentation of video clips, music and spoken stories, may be more effective in capturing the attention of participants compared to event-related paradigms (Bezdek et al., 2017), and are preferable for emotion induction (Joseph et al., 2020; Siedlecka & Denson, 2019).

To our knowledge, the first affective video databases were introduced by Philippot (1993) and Gross and Levenson (1995). After them, in order to enlarge such stimulus sets, many researchers began to compile their own. Schaefer et al. (2010) introduced FilmStim, a collection of 70 movie clips designed for the purpose of inducing emotional responses in experimental psychology studies. Jenkins and Andrewes (2012) presented a dataset of verbal and non-verbal contemporary films. The DEAP database, which was created by Koelstra et al. (2012), is a publicly available database consisting of 120 one-minute-long musical video excerpts. At least 14 volunteers using an online self-assessment tool that measures the levels of arousal, valence, and dominance induced by each clip rated these excerpts. Another noteworthy database is the MAHNOB-HCI, which was released by Soleymani et al. (2012). This multimodal database consists of 20 short emotional excerpts taken from commercially produced movies and video websites. The clips were carefully selected to represent a wide range of emotions and are accompanied by physiological data, including facial expressions and heart rate variability. Carvalho et al. (2012) also contributed to the field of emotional database creation by developing the Emotional Movie Database (EMDB). This database is composed of 52 non-auditory film clips, each lasting 40 seconds that were extracted from movies. The clips were selected to cover the entire affective space and were rated by 113 participants in terms of induced valence, arousal, and dominance using a nine-point scale. LIRIS-ACCEDE consists of 9,800 good quality video excerpts with a large content diversity (Baveye et al., 2015). Although they found a great amount of negative videos of high arousal, their positive videos induced largely “passive”, but not “active” arousal. In other words, this database formed a negative correlation between valence and arousal, which contradicts previous literature on the curvilinear relationship between these scales. Chieti Affective Action Videos is another database designed for the experimental study of emotions (Di Crosta et al., 2020). It consists of 360 videos of 15 seconds that depict only human actions, albeit in a good continuum of valence and arousal. “BioVid Emo DB” (Zhang et al., 2016) is another database, which contains not only self-reported data, but also skin conductance level, electrocardiogram, *trapezius* electromyogram of 86 subjects in response to affective videos.

In order to systematize all these properties and discrepancies between affective databases, Diconne et al. (2022) recently presented KAPODI, a searchable database of emotional stimuli sets in the tabular form. They found 24 databases of affective videos. However, only in nine of them were valence and arousal collected, and only six of them contained neutral videos. In two of the nine databases, 3 or 4 raters evaluated stimuli. Only in one database was the duration of videos fixed (2 s), while in others it ranged greatly (10 – 60 s or 25 – 161 s).

Overall, existing databases of emotion-eliciting video stimuli have different drawbacks. In many of them, the video duration varies, which does not permit researchers to select a sufficient number of equivalent videos (cropping videos is not appropriate since raters evaluated the whole videos in original databases). Speaking of cross-cultural studies, in many videos the voice is included that does not allow the use of it by scientists from other cultures. Though we admit that presenting the videos without audio can reduce the intensity of the emotions. In the current study, we decided to select silent (muted) video clips during preparation of the database. This was done in order to avoid cultural differences in the perception of voices and screams in different languages, situation nuances, faux pas and other language- or culture-specific affective factors.

We chose 1-minute excerpts in order to enrich stimulus material collection for neuroscientists. Thus, taking into account many central and peripheral variables, a one-minute epoch seems to fall into a range appropriate for many physiological systems. Thus, to begin with electroencephalography (EEG), the engagement index has been studied for epochs of one minute (Libert & Van Hulle, 2019), as well as valence and arousal indices of EEG (Xu et al., 2023). Many attempts on emotion recognition via EEG have been done using one-minute excerpts (Koelstra et al., 2012). Different metrics of subject synchronization, for example, inter-subject correlation of EEG, could be calculated for an epoch lasting for 1 minute (Dmochowski et al., 2014). Likewise, 1-minute stimuli can be used for inter-subject correlation calculations in fMRI paradigms within a standard range of temporal resolution (Imhof et al., 2017). In a neuroimaging study, it is essential to have repeated emotional experiences, but, at the same time, the procedure is generally constrained by time. Therefore, researchers require sufficiently long video clips to evoke and assess emotions each time, while ensuring they have enough time to allow for multiple samples of the same emotion. That is why, we believe, one-minute video clips could resolve the problem of duration/repetition (for example, a one-hour study can number about 30 – 45 video clips, that is 10 – 15 of each valence category, which may be enough for many designs).

As for the autonomic nervous system, Kreibig (2010) found that 1-minute epochs are the most popular segment type in studies of emotional responses. For instance, 1-minute intervals were found to be long enough in order to measure cardiac activity. Thus, Takahashi et al. (2017) found that low and high frequencies of heart rate variability could be extracted from one-minute epochs. In a study of Nussinovitch et al. (2011), RR intervals (intervals between two heartbeats) and root mean square of successive differences in RR intervals proved their reliability in 1-minute intervals. However, for such short periods, they did not recommend calculating standard deviation of the RR intervals and the proportion of intervals differing by 50 msec from the preceding interval. Skin temperature and skin conductance also could be studied for an interval of around 1 minute (Kosonogov et al., 2017). As for postural control, Carpenter et al. (2001) suggested that a sample duration of at least 60 s should be used to obtain a stable and reliable center of pressure characteristics.

## Method

First, six professional psychologists browsed the Internet (youtube.com, vk.com, videezy.com) in order to find each one independently 45 video clips (15 negative, 15 neutral, and 15 positive), approximately of one minute (they watched videos without sound). Video clips were muted one-minute excerpts from fiction, educational and documentary films or home movies. They represented a broad spectrum of plots, such as surgeries, suffering animals, starvation, and fights (negative), household, street and industrial scenes (neutral) and landscapes, joyful children, sports, romantic comedies (positive). This set of 270 videos was then evaluated by them on a three-point scale (negative/neutral/positive). Of them, 175 videos received the 100% inter-rater reliability (the same valence was assigned by all six raters). We then randomly selected 160 video clips (55 negative, 53 neutral and 52 positive ones) which comprised the final database. The mean duration was 60.7 s, *SD* = 1.9 s, ranging in length from 46 to 65 seconds, 87% of videos were from 58 to 62 s, 74% of videos were from 59 to 61 s. As in many affective video databases, like DEAP, MANHOB-HCIU, and OPEN_EmoRec_II (Rukavina et al., 2015) and also in a moral video database (McCurrie et al., 2018), we intended to obtain at least 30 ratings (subjects) for each video clip. That is why, of these 160 video clips, we built eight separate video clip samples consisting of 20 clips each to present to the study subjects. In other words, each rater evaluated 20 video clips, while each video clip was assessed by 30 raters. Each of eight video clip sample contained supposedly pleasant, unpleasant and neutral clips in approximately equal proportion (from 6 to 8 of each valence, 20 in total). The order of video clip presentation was chosen at random, corrected to avoid consecutive presentation of more than two clips of the same emotion category (Kosonogov, 2020). Participants were asked to evaluate each video clip in terms of valence (where 1 meant *Very negative* and 9 meant *Very pleasant*) and arousal (where 1 meant “*Very calm*” and 9 meant “*Very arousing*”) immediately after the presentation of each video clip. There was a break of 12 seconds between video clip presentations to minimize carry-over effects. The experimental paradigm was created with the use of *PsychoPy* software and conducted using the *Pavlovia* platform (pavlovia.org).

From 20/09/2023 to 6/02/2024, 185 raters (74% female participants, *M*_age_ = 24.8 years, *SD* = 8.0 years) participated in the study. Participants were recruited through electronic advertisements on the *VK* social network (they read instructions in Russian language and reported to live in Russia). Fifty-three participants took part in the study twice and one participant — three times: all recurring participants evaluated different video clip samples to avoid reassessments. Between-subject design was implemented: each person was randomly assigned to the group evaluating one 20-clip sample. All expressed the informed consent, were warned about violent content and blood in the scenes and were granted an equivalent of 5.5 USD (at purchasing power parity). The university ethical committee approved the study (Nº 92, 19.09.2022).

## Results

The mean of valence was 4.99, *SD* = 1.60, min = 1.33, max = 7.80; while the mean of arousal was 5.13 *SD* = 0.98, min = 2.63, max = 8.0. Fifty-five videos resulted to be negative, mean valence = 3.09, *SD* = 0.65, mean arousal = 5.60, *SD* = 0.90; 53 were neutral, mean valence = 5.25, *SD* = 0.50, mean arousal = 4.39, *SD* = 0.83, and 52 were positive, mean valence = 6.72, *SD* = 0.48, mean arousal = 5.38, *SD* = 0.74. Apparently, the analysis of variance revealed that positive videos were more positive than neutral, and neutral were more positive than negative ones, *F*(2,104) = 542.6, *p* < .001 (all post hoc test *p*s < .001). What is more important, negative and positive videos provoked greater arousal than neutral ones, *F*(2,104) = 31.8, *p* < .001, both post hoc test *p*s < .001, but the arousal between negative and positive videos did not differ, post hoc test *p* = .37.

**Figure 1:**
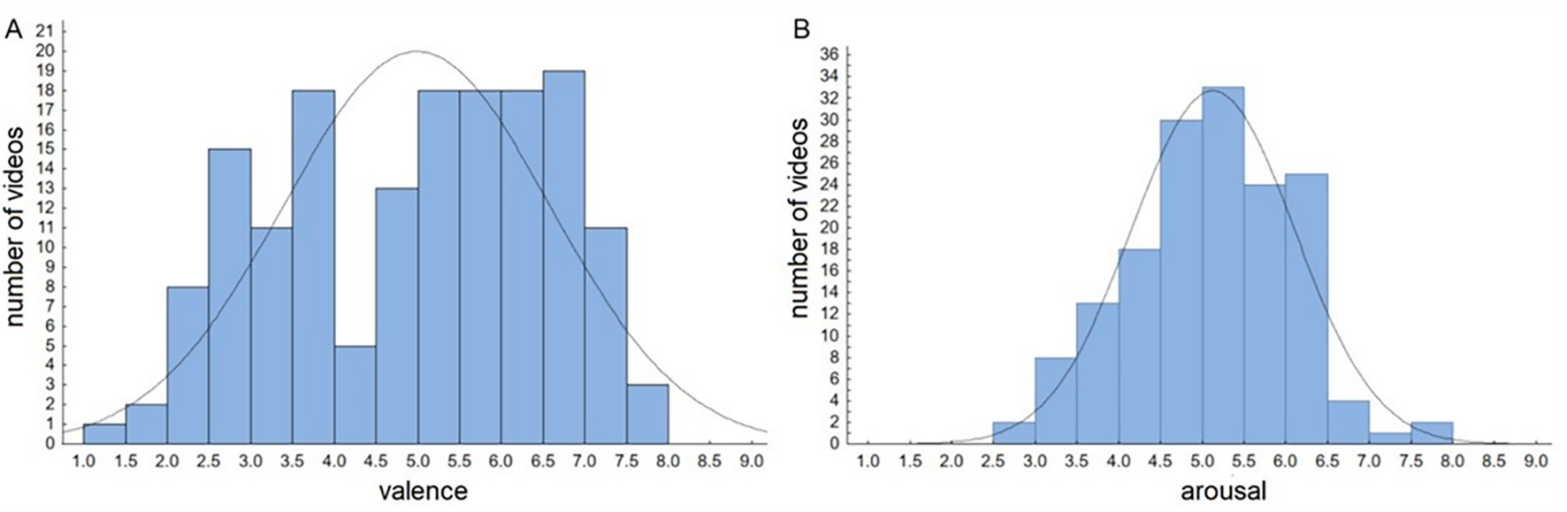
The Distribution of (A) Valence and (B) Arousal of Affective Videos

As for valence distribution, skewness (-0.27) and kurtosis (-1.05) were small, while a Kolmogorov-Smirnov test showed a non-normal distribution, *W* = .092, *p* < .001. Arousal turned out to be distributed normally, skewness = -0.03, kurtosis = -0.13, *d =* .057, *p =* .53 (Figure 1).

**Figure 2:**
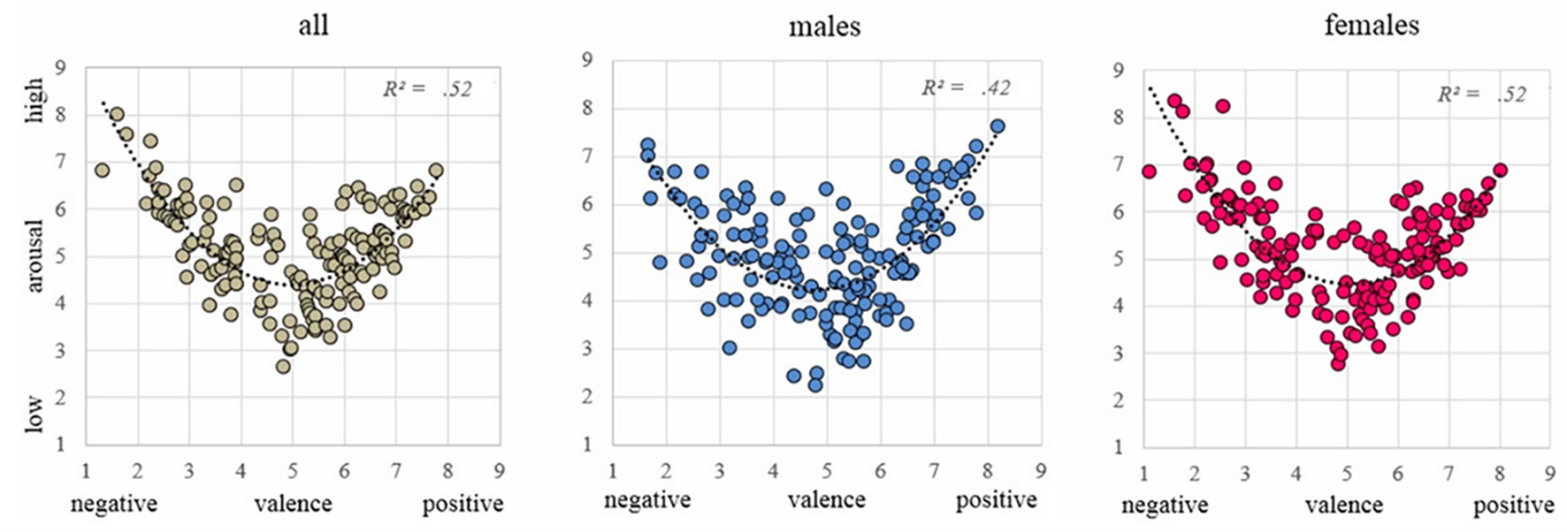
The Relationship Between Valence and Arousal of Affective Videos for the Three Categories of All Subjects, Males, and Females

The relationship between valence and arousal showed a typical quadratic pattern, *F*(2,157) = 87.66, *p* < .001, *R*^2^ = .52 (Figure 2). The same patterns were observed for the male, *F*(2,157) = 57.31, *p* < .001, *R*^2^ = .42, and the female subsamples, *F*(2,157) = 87.13, *p* < .001, *R*^2^ = .52. The internal consistency was questionable for valence (.60), while very good for arousal (.90). Correlations between means and standard deviations were significant. Means and *SD*s of valence correlated negatively, *r* = -.19, *p* = .017, while in the case of arousal the correlation was positive, *r* = .45, *p* < .001.

## Discussion

The presented dataset provides normative values of valence and arousal of 160 one-minute affective video clips. Each of the videos was evaluated by 30 subjects, while each subject evaluated at least 20 videos. The advantages of our dataset, in comparison to previous attempts to collect affective videos, are the following. First, the high number of videos of different valence categories allows researchers to compile appropriate subsets for their studies. Second, the equal and conventional duration of videos makes possible the usage of these videos in psychophysiological studies of many types (EEG, fMRI, peripheral polygraphy, posturography, TMS, etc.). Third, we excluded any sound or speech which would provoke any culture-dependent interpretation (e.g., language jokes), thus making our database useful in different countries.

As in many previous studies, the relationship between valence and arousal showed a typical quadratic pattern, that is, very negative and very positive videos were assessed with higher levels of arousal. Neutral videos received the lowest arousal scores. However, we admit that the lowest value of arousal was 2.63 (1 being the theoretical minimum over the scale from 1 to 9), while, for example, in the E-MOVIE database the lowest value of arousal was 1.9 (Maffei & Angrilli, 2019). We suppose that the mere situation of watching videos from different categories maintain the arousal level higher than 1.

Our videos were distributed along a quadratic curve, typical for valence-arousal relationship. Many other video databases also found this pattern (e.g., Ack Baraly et al., 2020). Curiously, some databases did not reveal a quadratic relationship, LIRIS-ACCEDE (Baveye et al., 2015) as well as in Gabert-Quillen et al. (2015), valence and arousal also correlated negatively, meaning that pleasant videos were perceived as less arousing. In line with a broad spectrum of studies, conducted with other types of affective stimuli such as pictures (Lang et al., 2008), sounds (Soares et al., 2013), and odors (Toet et al., 2020), we believe that the quadratic pattern found in our study reflects the nature of the relationship between valence and arousal of affective videos.

It is worth noting that the internal consistency (Cronbach’s alpha) for valence was questionable, while it was very good for arousal. This may indicate the high ambivalence associated with some highly affective stimuli. Additionally, we highlight that the standard deviation for valence was higher compared to arousal, contrary to findings from E-MOVIE (Maffei & Angrilli, 2019), where arousal displayed less variability than valence. In simpler terms, the videos in our dataset were generally seen as arousing stimuli, but their valence ratings were less certain on average. Some neutral video clips received high arousal ratings, which may reflect not the neutral nature of these stimuli, but rather a mixture of both negative and positive emotions. Future studies could employ not the unique valence scale (from negative to positive), but two distinct scales of negativity and positivity of each stimulus (Ito & Cacioppo, 2005). Such an evaluation could identify ambivalent stimuli which can be important for researchers to select or avoid them, depending on the purposes. We also examined the correlation between means and standard deviations. There was a weak negative correlation between the means and *SD*s of valence, that is for negative videos *SD* was higher. The means and *SD*s of arousal displayed a moderate positive correlation, which aligns with the OASIS database (Kurdi et al., 2017).

Curiously, several negative videos received greater arousal scores than the most positive. Three negative videos provoked arousal scores greater than 7, while no positive video was perceived with an arousal score higher than 7. Also, the most negative videos were very close to the negative pole (valence < 2), while there was no video which received a valence score greater than 8. This seems to reflect an effect, called negativity bias, (Ito et al., 1998) that lies in the fact that the most unpleasant pictures evoke greater emotional reactions than the most pleasant. In other words, threatening stimuli typically provoke faster and larger reactions because evolution processes favored those types of behavior which are related to survival, in comparison to behavior in response to neutral or pleasant stimuli.

Nevertheless, our database encompasses more than 50 videos of each category (negative, neutral, and positive). Taking into account that the duration of future experiments and the number of videos to be presented are limited, we believe our database will permit researchers to select corresponding subsamples of videos from different categories.

## Supplementary Materials

**Table:** 

Type of supplementary materials	Availability/Access
Data	
a. Silent video clips.	Kosonogov (2024)
b. S-Table 1 contains each video's averaged valence and arousal with duration and source.	Kosonogov (2024)
c. S-Table 2 contains raw data of each rater.	Kosonogov (2024)

## Data Availability

The dataset for this study can be found at Kosonogov (2024). S-Table 1 contains the averaged valence and arousal for each video with its duration and source. S-Table 2 contains the raw data of each rater. The videos can be found via links or upon request.
